# Supplementary material for: Feasibility and Preliminary Efficacy of Wearable Focal Vibration Therapy on Gait and Mobility in People with Multiple Sclerosis: A Pilot Study
Source: Bioengineering (Basel). 2025 Aug 29;12(9):932. doi: 10.3390/bioengineering12090932 (PMC12467686; doi:10.3390/bioengineering12090932)
Supplement: Supplementary file 1 [file bioengineering-12-00932-s001.zip › bioengineering-3832053-supplementary.pdf]

MYOVOLT™

USER MANUAL

WEARABLE VIBRATION THERAPY

MYOVOLT is a new Wearable Technology that applies *Localised Vibration Therapy* to any part of the body. MYOVOLT treats muscles and joints to help you work harder, move better and recover faster.

PERFORM BETTER
Super-charge your warm-up to boost power, train harder and avoid injury.

RECOVER FASTER
Reduce soreness. Speed-up recovery of muscles and joints to feel better, faster.

MOVE EASIER
Relax muscles to improve flexibility and range of motion.

READY TO WEAR
Wearable anywhere for sports and fitness therapy.

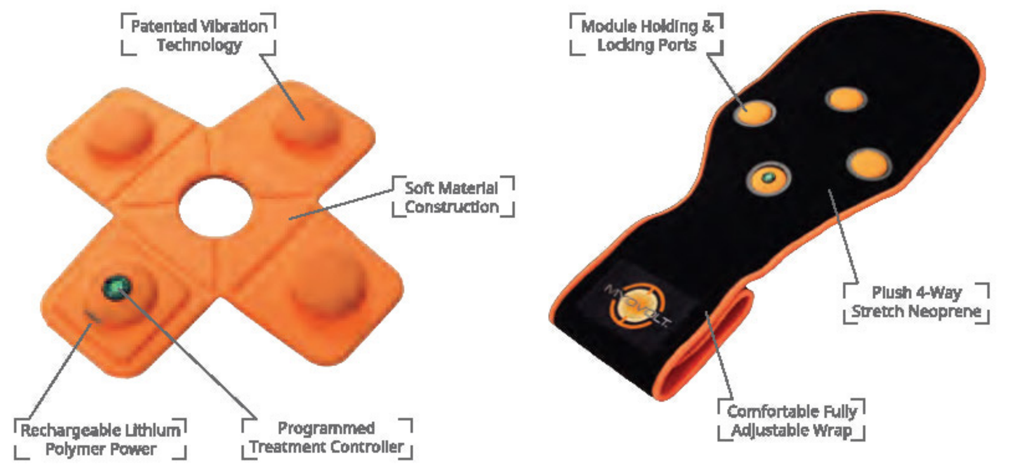

THE TECHNOLOGY

MYOVOLT is the world's first clinically proven wearable technology using Localized Vibration Therapy for muscle performance & recovery.

MYOVOLT delivers vibration frequencies known to stimulate muscles, increase circulation, flexibility, range of motion and relieve soreness.

Wear MYOVOLT to massage muscles before or after your workout or sports training to reduce tightness, ease pain and recover faster.

Our breakthrough wearable design targets vibration therapy on any part of the body and is comfortable to wear whilst moving around. MYOVOLT can be worn over the top of clothing or next to skin.

With digital control, rechargeable battery and unique flexible design its easy to use, very lightweight, comfortable and totally portable.

Sports massage where and when you need it.
WEAR YOUR THERAPY

WEARING MYOVOLT PRO

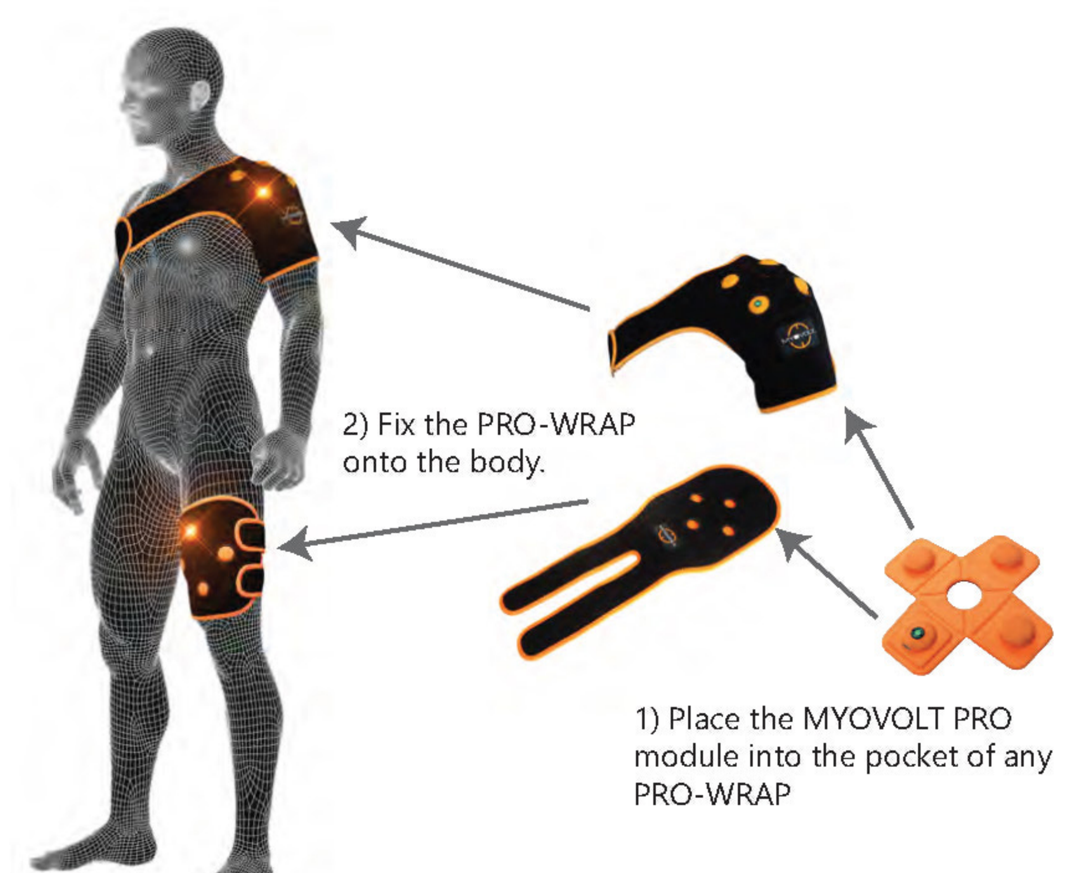

For more instructions go to:
http://www.myovolt.com/use

WEARING MYOVOLT CORE

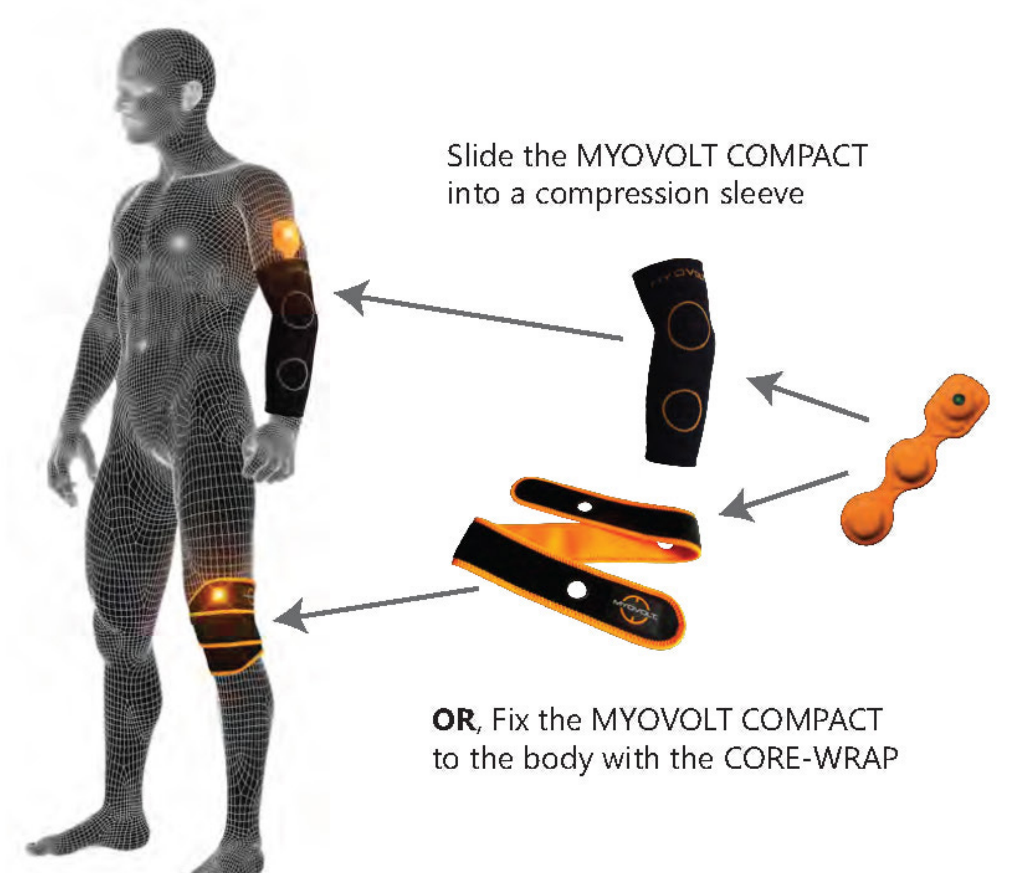

For more instructions go to:
http://www.myovolt.com/use

QUICK START

To turn ON the MYOVOLT press the CONTROL switch for 2 secs.
The MYOVOLT has three vibration modes. Press the switch Once to change between the modes.

BATTERY LEVEL METER

Press the CONTROL switch at any time to check the battery power level.

70-100% power remaining (green)
30-70% power remaining (orange)
<30% power remaining (red)

CONTROL MENU

Switch Press	Function	LED indicator
Press Once (2 sec hold)	Turn Myovolt on Pulse mode	Wave pulse orange light
(short press)	Alternating mode	Flashing orange light
(short press)	Constant Mode	Continuous orange light
Press and Hold 2 secs any time	*Turn MYOVOLT OFF*	LED off

MYOVOLT MODULE
USB Port and Control Switch

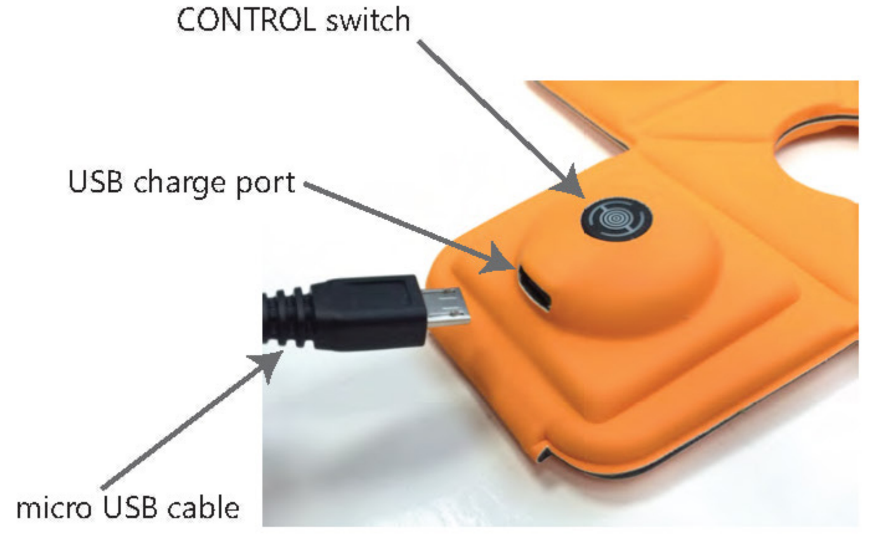

OPERATING INSTRUCTIONS

read before use

CHARGING

• To charge the module plug the micro USB cable into the USB charge port
• A red light will display while charging and turn green when fully charged (~3hours)
• While the module is switched off you can Press the Power Switch to check the battery level. Green >70% charge, Orange 30% - 70% charge, Red <30% charge

OPERATION

• Press and hold the power button once to turn module on to **program one**, twice to turn module to **program two** and three times for **program three**. Pressing the power button again will turn the unit off. The Orange power light shows the unit is on and coresponds to the vibration program setting.

• Press and hold at any time to turn the module off. The module will turn off automatically after **10 minutes** of use. To continue use, simply press the power button again to restart.

• Each treatment cycle runs for 10mins. Use for One or Two treatment cycles per day on a selected body area.

CLEANING & MAINTENANCE

• The Myovolt module can be cleaned with a moist towel.
• Dont let moisture enter the CONTROL switch or charging port
• Keep unit away from heat sources, liquids, and children

SPECIFICATION

• 3x (or 2x) 2.5W frequency tuned actuator motors
• Built-in rechargeable 500-800mAh lithium-polymer battery
• Power level meter, USB recharge control
• Charge using any USB source or charger
• Charge time: ~ 3hrs
• Charge V: USB 5V
• Running time: up to 100mins continuous run time

CAUTIONS and WARNINGS

• Do not immerse vibration module in water. Keep liquids away from the switch and charge port
• Do not attempt to disassemble.
• Do not use for more than 30mins per day on the same body location.
• Unplug the module after fully charged
• Store in a cool dry location when not in use
• For use by adults only, keep out of reach of children
• Consult your doctor or physio before treating any serious injury or if pain persists.
• Do not over heat or store in a hot area
• Use product only according to provided instructions
• Warning: this device should not be used over swollen or inflamed areas or skin eruptions.

DISCLAIMER

PLEASE DO NOT USE MYOVOLT WITHOUT FIRST CONSULTING WITH YOUR DOCTOR IF ANY OF THE FOLLOWING APPLY:
Pregnancy, diabetes with complications such as neuropathy or retinal damage, using pace-makers, recent surgery, epilepsy, migraines, herniated disks, spondylolisthesis, spondylolysis, or spondylosis, recent joint replacements or IUD's, metal pins or plates or any concerns about your physical health.

1 YEAR WARRANTY

If within one year from the date of purchase, this product fails due to a defect in material or workmanship, MYOVOLT or its International distributor/retailer where the product was purchasded will repair or replace the product free of charge.

This warranty excludes:
1) Damage caused by accident, abuse, mishandling, or transport
2) Products subject to unauthorised repair
3) Products used not in accordance with provided instructions
4) Damage exceeding the cost of the product
5) Damage caused by normal wear and tear
5) Deterioration of the delivered product resulting from abnormal storage
6) Failure to provide a dated proof of purchase

Some states and countries do not allow a limitation of damages, so the foregoing limitation may not apply to you. This warranty guarantees specific legal rights, and but other rights may vary from country to country and from state to state. This warranty service is available through the operating policies and procedures of MYOVOLT LIMITED, and all its international distributors. If you are a client, please contact the appropriate MYOVOLT business entity, dealer or retailer, from whom you directly purchased the product for warranty and return authorization procedures. This warranty is initiated and executed by MYOVOLT LIMITED.

CONTACT

info@myovolt.com

MYOVOLT LIMITED
146a Lichfield St
Christchurch
New Zealand

MYOVOLT™

Designed in New Zealand, assembled in China
